# Effect of Bias Voltage on Mechanical Properties of HiPIMS/RFMS Cosputtered Zr–Si–N Films

**DOI:** 10.3390/ma12172658

**Published:** 2019-08-21

**Authors:** Yung-I Chen, Yu-Zhe Zheng, Li-Chun Chang, Yu-Heng Liu

**Affiliations:** 1 Institute of Materials Engineering, National Taiwan Ocean University, Keelung 20224, Taiwan; 2 Center of Excellence for Ocean Engineering, National Taiwan Ocean University, Keelung 20224, Taiwan; 3 Department of Materials Engineering, Ming Chi University of Technology, New Taipei 24301, Taiwan; 4 Center for Plasma and Thin Film Technologies, Ming Chi University of Technology, New Taipei 24301, Taiwan

**Keywords:** bias voltage, elastic recovery, HiPIMS, H/E*, H^3^/E*^2^, mechanical properties, residual stress, RFMS

## Abstract

Zr–Si–N films with atomic ratios of N/(Zr + Si) of 0.54–0.82 were fabricated through high-power impulse magnetron sputtering (HiPIMS)–radio-frequency magnetron sputtering (RFMS) cosputtering by applying an average HiPIMS power of 300 W on the Zr target, various RF power levels on the Si target, and negative bias voltage levels of 0–150 V connected to the substrate holder. Applying a negative bias voltage on substrates enhanced the ion bombardment effect, which affected the chemical compositions, mechanical properties, and residual stress of the Zr–Si–N films. The results indicated that Zr–Si–N films with Si content ranging from 1.4 to 6.3 atom % exhibited a high hardness level of 33.2–34.6 GPa accompanied with a compressive stress of 4.3–6.4 GPa, an H/E* level of 0.080–0.107, an *H*^3^/E*^2^ level of 0.21–0.39 GPa, and an elastic recovery of 62–72%.

## 1. Introduction

Nanocomposite Zr–Si–N films have attracted considerable research interest because of their mechanical properties and oxidation resistance; these films with 2–6 atom % Si exhibit a hardness level of 30–36 GPa [1,2,3,4], whereas the films with a high Si content (15–30 atom %) display remarkable oxidation resistance at 600 °C accompanied with a low hardness level of 12–16 GPa [5,6]. An nc-M_n_N/a-Si_3_N_4_ model has been developed to estimate the hardness improvement of low-Si-content nanocomposite M–Si–N films (M: transition metal) [7,8,9,10,11]. Nanocrystalline M–N grains surrounded by an amorphous Si_3_N_4_ matrix improve the film hardness attributed to strengthening mechanisms including solid-solution strengthening, nanocomposite formation hardening, and residual stress effect [11]. However, in contrast to Ti–Si–N films, the hardness improvement of Zr–Si–N films by adding Si to form nanocomposite films is limited [1,2,3,4,5]. High-power impulse magnetron sputtering (HiPIMS) with a dense plasma comprising a high degree of ionization of the sputtered materials [12,13,14] was used to fabricate films with a dense structure accompanied with a high hardness and high residual compressive stress [15,16]. Moreover, hybrid processes such as direct-current magnetron sputtering (HiPIMS-DCMS) [17] and radio-frequency magnetron sputtering (HiPIMS-RFMS) [18] were used to increase the deposition rate, which was a limitation of the conventional HiPIMS process [13,14]. In a previous study [19], the crystalline Zr–Si–N films of 2–6 atom % Si prepared using a HiPIMS-RFMS process showed low surface roughness (0.8–1.4 nm) and high nanoindentation hardness (33.1–34.3 GPa), Young’s modulus (346–373 GPa), and compressive residual stress (4.4–5.0 GPa). Tang et al. [20] fabricated Zr–Si–N films through a hybrid system comprising a superimposed HiPIMS-medium frequency and RF sputtering at a 200 °C substrate temperature and −100 V bias voltage; their results exhibited that the films containing 4.7 atom % Si had the highest hardness (33.4 GPa). Moreover, in our previous study [19], the hardness and Young’s modulus of the HiPIMS-RFMS cosputtered Zr–Si–N films exhibited linear relationships to their compressive residual stresses ranging from −0.2 to −5.0 GPa. Therefore, it is crucial to explore increasing the mechanical properties of Zr–Si–N films, and hence, its compressive residual stress was increased through application of a negative bias voltage on the substrates during deposition. In fabricating binary nitride and carbide films through the sputtering processes, applying a negative bias voltage on substrates increased the kinetic energy of the bombarding positive ions, which resulted in the increase in the adatom mobility (ion-bombardment-enhanced diffusion), resputtering, and atomic peening [21,22,23]. The ion bombardment peening affected the residual stress and hardness [24]. Moreover, it induced complete phase separation of the nanocomposite M–Si–N films [25] and resputtered the light Si adatoms [26]. In this study, the residual stress of Zr–Si–N films was fabricated through the HiPIMS-RFMS hybrid process, which was increased to 8.8 GPa in compression by applying a substrate bias voltage of −150 V. The effects of substrate bias voltage on the chemical compositions, phase structures, and mechanical properties of Zr–Si–N films were investigated.

## 2. Materials and Methods 

The HiPIMS-RFMS cosputtering system was illustrated in detail in a previous study [19]. The Ti and Zr targets with a diameter of 76.2 mm were connected to a pulse power supply (SPIK 2000A, Shen Chang Electric, Taipei, Taiwan), whereas the Si target with a diameter of 50.8 mm was connected to a radio-frequency power generator of 13.56 MHz. The substrate-to-target distance was 12 cm. The rotating (10 rpm) substrate holder without heating was maintained at less than 70 °C during deposition. A Ti interlayer was deposited on silicon wafers at 400 W for 40 min under a working pressure of 0.4 Pa at a steady flow of 30-sccm Ar. Zr–Si–N films were cosputtered on Ti interlayers after flowing a gas mixture of Ar and N_2_ set at 28 and 2 sccm, respectively, into the chamber, and the working pressure was maintained at 0.4 Pa. An average HiPIMS power of 300 W was applied on the Zr target, and batches A, B, and C were prepared using RF powers of 30, 40, and 50 W, respectively, on a Si target, and the substrate bias voltage was set at 0, −50, −100, and −150 V.

The chemical compositions of the films were analyzed using a field-emission electron probe microanalyzer (FE-EPMA, JXA-8500F, JEOL, Akishima, Japan). The Ti signal from interlayers was not detected, which implied that no Si signal from substrates was detected. The film thickness was evaluated through field emission scanning electron microscopy (FE-SEM, S4800, Hitachi, Tokyo, Japan). The phases of the films were analyzed using an X-ray diffractometer (XRD, X’Pert PRO MPD, PANalytical, Almelo, The Netherlands) with Cu Kα radiation through the grazing incidence technique with an incidence angle of 1°. Moreover, the XRD patterns for determining the texture coefficients were measured following a Bragg–Brentano scan. The surface roughness values of the films were evaluated using an atomic force microscope (DI 3100, Bruker, Santa Barbara, CA, USA). The scanning area of each image was set at 5 μm × 5 μm at a scanning rate of 1.0 Hz. The hardness (*H*) and elastic modulus (*E*) values of films were measured using a nanoindentation tester (TI-900 Triboindenter, Hysitron, MN, USA) equipped with a Berkovich diamond probe tip. The indentation depth was 80 nm. The *H* and effective Young’s modulus E* (E* = *E*/(1 − *ν*^2^)) values were calculated based on the Oliver and Pharr method [27], where *ν* is the Poisson ratio and is set at 0.31. The elastic recovery, *We*, was determined from the loading–unloading curves measured using nanoindentation testing [28]. The residual stress of the films was calculated using Stoney’s equation [29]. The measurements on the film curvatures were calibrated using BK7 glass plates with curvatures of 0, −0.1, and +0.1 m^−^^1^.

## 3. Results and Discussion

### 3.1. Chemical Compositions and Phases

**Figure 1:**
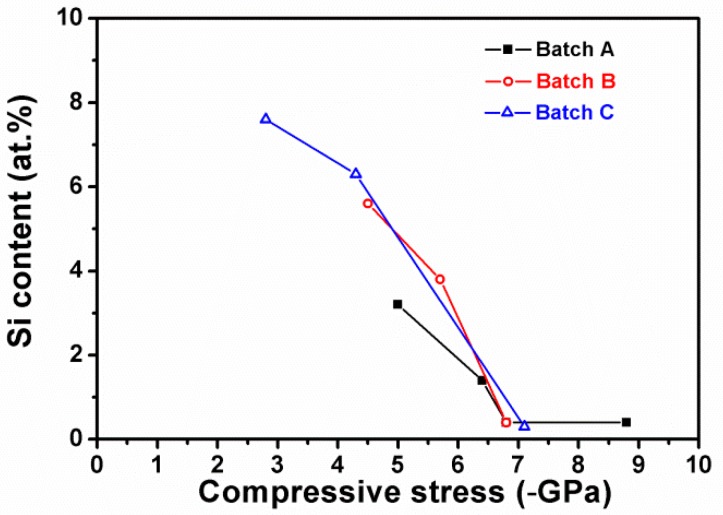
Relationship between Si content and residual stress of Zr–Si–N films.

Table 1 presents the chemical compositions of Zr–Si–N films fabricated using an average HiPIMS power (P_Zr_) of 300 W on the Zr target, various RF powers (P_Si_) applied on the Si target, and negative bias voltage levels of 0–150 V connected to the substrate holder. The Zr–Si–N films were denoted as Zr_x_Si_y_N_100__−__x−__y_ after ignoring the O content. All the Zr–Si–N films exhibited an atomic ratio [N/(Zr+Si)] of 0.54–0.82, which was below the stoichiometric ratio of 1.0 for ZrN. The Si content of the films in batch A decreased from 3.2 to 1.4 and 0.4 atom %, whereas the N content increased from 34.9 to 36.2 and 37.3 atom % when the negative bias voltage level was increased from 0 to 50 and 100 V, respectively. The films prepared at bias voltages of −100 V and −150 V were Zr_62.3_Si_0.4_N_37.3_ and Zr_62.2_Si_0.4_N_37.4_, respectively, which exhibited a constant and Si-less level attributed to severe ion bombardment. In our previous study on HiPIMS-RFMS-prepared Zr–Si–N films fabricated using the same gas flow (Ar: 28 sccm, N_2_: 2 sccm) at ground state [19], the Si content increased from 0 to 10 atom % accompanied with an increase in N content from 29 to 40 atom %, which was attributed to the high affinity of Si and N [11,20]. Moreover, the Si content of the samples in batches B and C exhibited decreasing tendencies with an increase in substrate bias voltage, whereas the variation in N content was not correlated with the substrate bias voltage. Furthermore, the compressive residual stress increased with an increase in the substrate bias voltage for all the three batches. Therefore, applying a negative bias voltage resulted in a decrease in Si content (Figure 1), which was attributed to the ion bombardment and resputter effect [26]. All the films prepared at a substrate bias voltage above −100 V exhibited a negligible Si content of 0.3–0.4 atom %. The deposition rates slightly decreased when a substrate bias voltage was applied. The film thickness levels were controlled at 747–1080 nm through adjustment of the deposition time after considering an indentation depth of 80 nm for evaluating the films’ mechanical properties.

**Figure 2:**
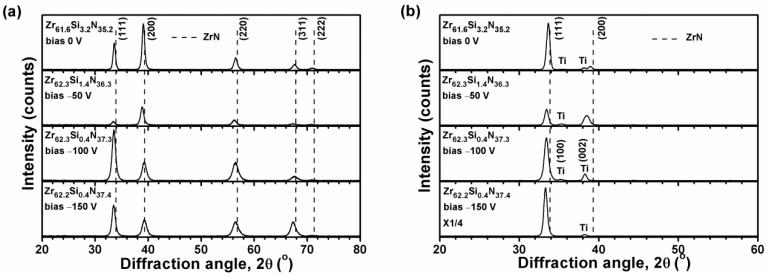
(**a**) Grazing incidence (GIXRD) and (**b**) Bragg–Brentano XRD patterns of the Zr–Si–N films prepared using P_Zr_ = 300 W and P_Si_ = 30 W on Si substrates.

**Figure 3:**
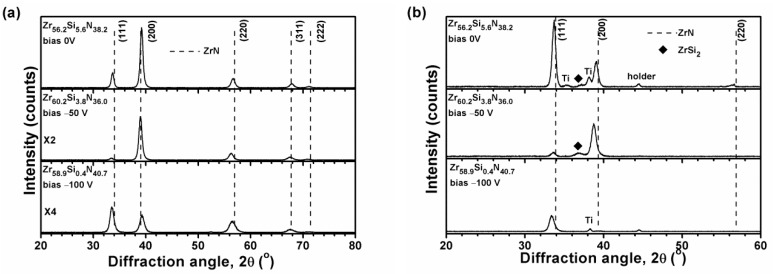
(**a**) GIXRD and (**b**) Bragg–Brentano XRD patterns of the Zr–Si–N films prepared using P_Zr_ = 300 W and P_Si_ = 40 W on Si substrates.

**Figure 4:**
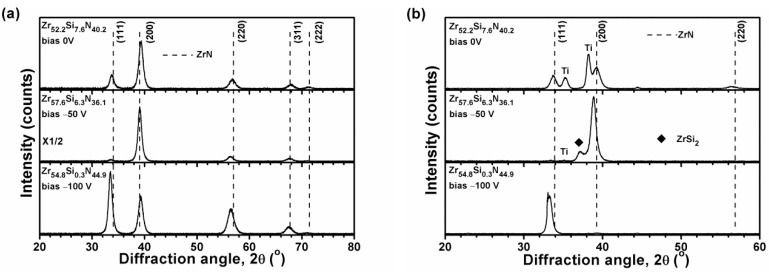
(**a**) GIXRD and (**b**) Bragg–Brentano XRD patterns of the Zr–Si–N films prepared using P_Zr_ = 300 W and P_Si_ = 50 W on Si substrates.

**Figure 5:**
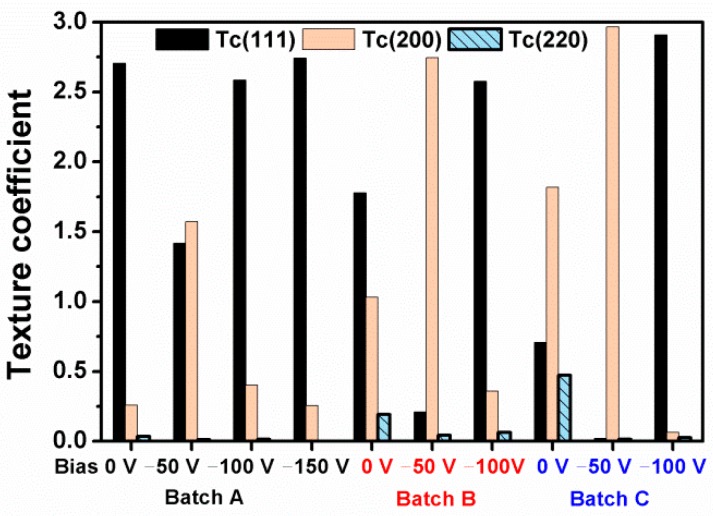
Texture coefficients of Zr–Si–N films.

**Figure 6:**
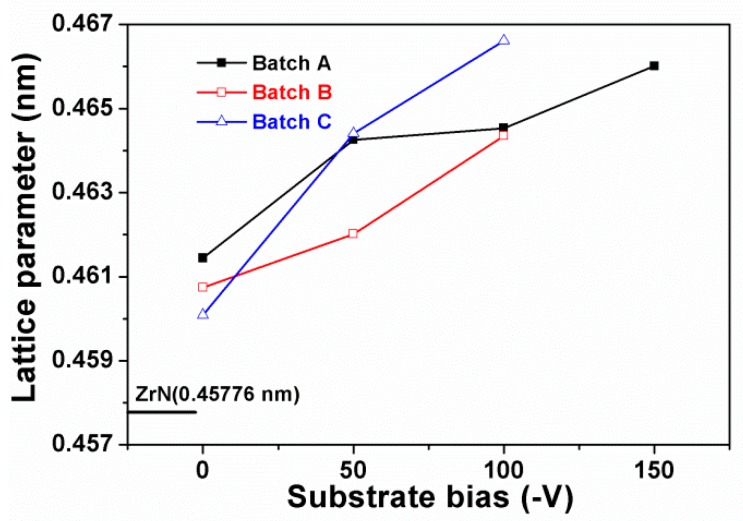
Lattice parameters of Zr–Si–N films fabricated under various substrate holder bias voltages.

Figure 2a, Figure 3a, and Figure 4a illustrate the grazing incidence XRD (GIXRD) patterns of the films in batches A, B, and C, respectively, which display a face-centered cubic (fcc) ZrN [ICDD 00-035-0753] phase. In our previous study [19], the HiPIMS-RFMS-fabricated Zr–Si–N films with Si content less than 7.6 atom % were crystalline, whereas Zr–Si–N films with Si content more than 10 atom % had a dominant X-ray amorphous phase. Figure 2b illustrates the XRD patterns of the Zr–Si–N films in batch A obtained through Bragg–Brentano scan. The fcc (111) and (200) reflections shifted toward the left side while raising the negative bias voltage level from 0 to 150 V. The shifted ZrN (200) reflection of the Zr_62.3_Si_1.4_N_36.3_ films overlapped with a Ti (002) signal. The standard intensity ratio of I_(111)_:I_(200)_:I_(220)_ for an fcc ZrN phase is 100:74:36. Figure 5 shows the texture coefficients *Tc* [23] of the Zr–Si–N films calculated using (111), (200), and (220) reflection intensities. The orientation of batch A films varied from (111) for the Zr_61.6_Si_3.2_N_35.2_ films prepared at a ground voltage level to (200) for the Zr_62.3_Si_1.4_N_36.3_ films prepared at a bias voltage of −50 V and then back to (111) for the Zr_62.3_Si_0.4_N_37.3_ and Zr_62.2_Si_0.4_N_37.4_ films prepared at a bias voltage of −100 and −150 V, respectively. Similar variations were observed for the Bragg–Brentano XRD patterns of the films in batches B and C as shown in Figure 3b and Figure 4b. The orientations of batch B films were (111), (200), and (111) for the films prepared at a bias voltage of 0, −50, and −100 V, respectively. The orientation of batch C films was (200) for the films prepared at a bias voltage of 0 and −50 V, whereas the orientation was (111) for the films prepared at a bias voltage of −100 V. The ZrSi_2_ phase [ICDD 00-032-1499] was observed besides a ZrN phase for the films in batches B and C. Because the standard Gibbs free energy levels of ZrN, Si_3_N_4_, and ZrSi_2_ at 298 K are −673.398 kJ/mol of Zr or N, −161.836 kJ/mol of N, and −157.931 kJ/mol of Zr [30], respectively, ZrN form preferentially, and excess Zr or N bind to Si. Moreover, the standard Gibbs free energies of Si_3_N_4_ and ZrSi_2_ were also interpreted as −215.781 and −78.966 kJ/mol of Si, respectively, which implies that Si_3_N_4_ is more stable than ZrSi_2_. However, the surveyed Zr–Si–N films in this study exhibited high Zr and low N contents, which caused the formation of major ZrN and minor ZrSi_2_ phases. Sandu et al. [11,31] proposed a solubility limit of 4 atom % Si for Zr–Si–N films, and the ZrN grain size and Si solubility limit decreased by applying bias voltage. In our previous study [19], the HiPIMS-RFMS-fabricated Zr–Si–N films exhibited a Si solubility of 5.6 atom %. The three Si-less films Zr_62.3_Si_0.4_N_37.3_, Zr_58.9_Si_0.4_N_40.7_, and Zr_54.8_Si_0.3_N_44.9_ prepared with a substrate bias voltage of −100 V revealed the characteristics of ZrN films deposited through strong ion bombardment, which exhibited a strong (111) texture [15]. Figure 6 displays the lattice parameters of the Zr–Si–N films calculated using (111) reflections in Bragg–Brentano XRD patterns. The lattice parameters increased with an increase in the substrate bias voltage and were larger than the standard value of 0.45776 nm for fcc ZrN, which implies that the films fabricated under a high substrate bias voltage exhibit a high residual stress during compression. Applying a substrate bias voltage resulted in a decrease in surface roughness to less than 2 nm (Table 1), possibly accompanied with structural densification [32,33].

**Figure 7:**
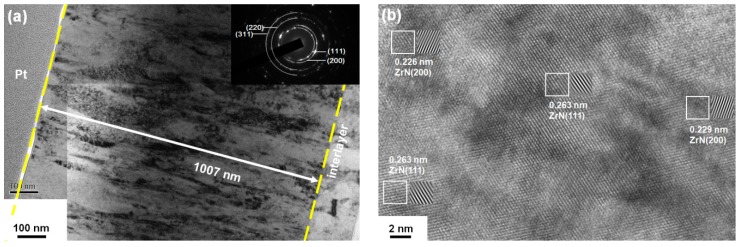
(**a**) Cross-sectional transmission electron microscopy (TEM) image and selected area electron diffraction (SAED) pattern and (**b**) high-resolution TEM image of the Zr_62.3_Si_1.4_N_36.3_ films (batch A).

**Figure 8:**
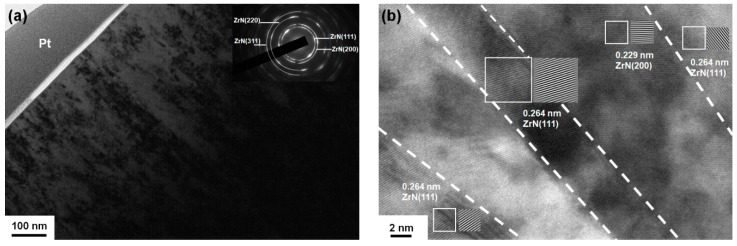
(**a**) Cross-sectional TEM image and SAED pattern and (**b**) high-resolution TEM image of the Zr_54.8_Si_0.3_N_44.9_ films (batch C).

The cross-sectional transmission electron microscopy (TEM) image of the Zr_61.6_Si_3.2_N_35.2_ films (batch A) prepared at a ground voltage level was displayed in a previous study [19], which exhibited a columnar structure. Figure 7a displays the cross-sectional TEM image of the Zr_62.3_Si_1.4_N_36.3_ films (batch A) prepared at a bias voltage of −50 V, which exhibits a crystalline structure. Figure 8a displays the cross-sectional TEM image of the Zr_54.8_Si_0.3_N_44.9_ (batch C) prepared at a bias voltage of −100 V, which exhibits a crystalline structure. Figure 7b and Figure 8b exhibit lattice fringes of crystalline ZrN regions. All the films, namely Zr_61.6_Si_3.2_N_35.2_, Zr_62.3_Si_1.4_N_36.3_, and Zr_54.8_Si_0.3_N_44.9_, were crystalline, and amorphous Si_3_N_4_ regions were not observed, which indicated that Si atoms substituted Zr atoms in the ZrN lattice as the Si contents were lower than a solubility limit [11].

### 3.2. Mechanical Properties

**Figure 9:**
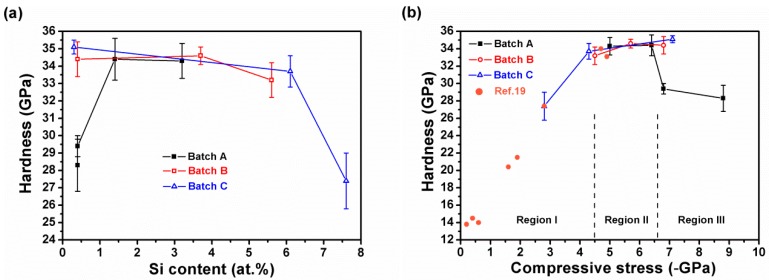
Relationships between nanoindentation hardness and (**a**) Si content and (**b**) residual stress of Zr–Si–N films.

Table 2 presents the *H*, E*, H/E*, and *H*^3^/E*^2^ values of the Zr–Si–N films. Figure 9a illustrates the nanoindentation hardness values of the Zr–Si–N films with various Si contents. The films with Si content of 1.4–6.3 atom % exhibited a high hardness level (33.2–34.6 GPa). Previous studies have reported a maximum hardness of 29.8–36 GPa accompanied with an Si content of 2–6.2 atom % for Zr–Si–N films [1,2,3]. The Zr_52.2_Si_7.6_N_40.2_ film, one of the batch C films, prepared without applying a substrate bias voltage exhibited a low residual stress of −2.8 GPa and showed a relatively low hardness of 27.4 GPa (Table 2). The Si content of batch C films decreased with an increase in substrate bias voltage due to the ion bombardment effect. Thus, Zr_57.6_Si_6.3_N_36.1_ films prepared at a bias voltage of −50 V exhibited an Si content in the range of 1.4–6.3 atom % accompanied with a high hardness (33.7 GPa). Moreover, the Zr_56.2_Si_5.6_N_38.2_ and Zr_60.2_Si_3.8_N_36.0_ films (batch B) prepared at a ground voltage level and a bias voltage of −50 V, respectively, exhibited an Si content of 3.8–5.6 atom % and high hardness levels of 33.2 and 34.6 GPa, respectively. Furthermore, similar results were observed for the Zr_61.6_Si_3.2_N_35.2_ and Zr_62.3_Si_1.4_N_36.3_ films (batch A), which exhibited high hardness levels of 34.3 and 34.4 GPa, respectively. By contrast, the three Si-less films, Zr_62.3_Si_0.4_N_37.3_ (batch A), Zr_58.9_Si_0.4_N_40.7_ (batch B), and Zr_54.8_Si_0.3_N_44.9_ (batch C), prepared at a substrate bias voltage of −100 V exhibited hardness values of 29.4, 34.4, and 35.1 GPa accompanied with residual stress levels of −6.8, −6.8, and −7.1 GPa, respectively, and the films with a higher N content exhibited higher hardness. In a previous study [34], the ZrN_x_ films (x = 0.65–0.78) prepared using the HiPIMS system at a −100 V substrate bias voltage and a substrate temperature of 400°C exhibited a hardness level of 26–27 GPa accompanied with a residual stress ranging from −4.2 to −5.2 GPa. The Zr_54.8_Si_0.3_N_44.9_ films exhibited a relatively high residual stress of −7.1 GPa and the highest hardness of 35.1 GPa in this study, which were comparable with the ZrN films fabricated through HiPIMS reported by Purandare et al. [15]; in their study, the samples prepared at bias voltages of −65, −75, and −95 V exhibited residual stresses of −5.1, −7.7, and −10 GPa and hardness values of 31.9, 36.6, and 40.4 GPa, respectively. Figure 9b illustrates the hardness values of Zr–Si–N films with various residual stresses, which includes some data from a previous study [19]. These data were divided into three categories according to their residual stress levels. The hardness levels of the HiPIMS-RFMS-cosputtered Zr–Si–N films exhibited a linear relationship with their residual stresses ranging from −0.2 to −4.5 GPa (Region I), whereas the hardness values maintained a similar level accompanied with a residual stress ranging from −4.5 to −6.4 GPa (Region II) and exhibited diversified values at a residual stress of more than −6.8 GPa (Region III). Mae et al. [1] reported that the hardness and stress exhibited a linear trend for the ZrSiN films with stress less than −5 GPa, which was a result of lattice distortion caused by the difference in the atomic sizes of Zr, Si, and N. By contrast, Qi et al. [35] reported that the hardness of ZrN coatings increased with an increase in residual stress up to 4.24 GPa in compression, and that further increasing the compressive stress to more than 7.95 GPa resulted in a decreasing trend in hardness, which was attributed to the inverse Hall–Petch effect.

**Figure 10:**
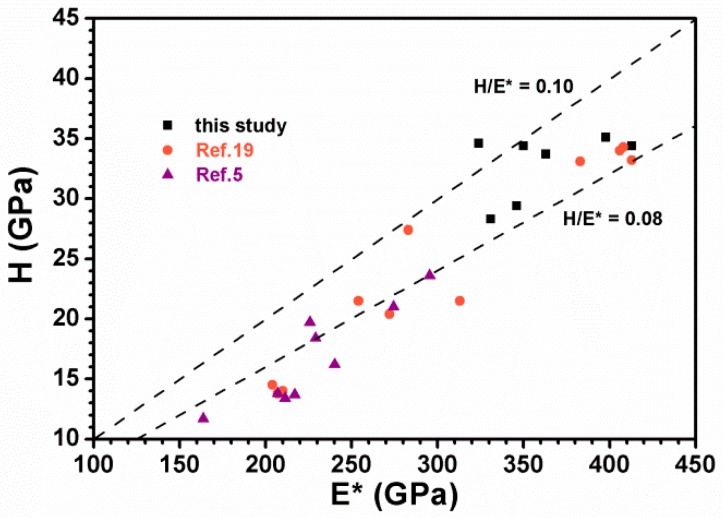
Relationship between hardness (*H*) and effective Young’s modulus (E*) of the Zr–Si–N coating.

The ratios of *H/E* [36,37,38] and H/E* [4,39] denote elastic strain to failure, which assisted to assess the wear resistance of hard coatings with the criteria of *H/E* > 0.1 [38] and H/E* > 0.1 [39]. Figure 10 depicts the relationship between *H* and E* in which the data of the batches A, B, and C prepared at the ground state were classified as reported in Ref. 19. The data of conventional DCMS-prepared Zr–Si–N films (Ref. 5) are also shown for comparison. The HiPIMS-RFMS-cosputtered Zr–Si–N films prepared at bias voltages of −50, −100, and −150 V exhibited a high H/E* level of 0.083–0.107 or a high *H/E* level of 0.092–0.118. By contrast, HiPIMS-RFMS-cosputtered Zr–Si–N films prepared without applying a negative substrate bias voltage exhibited median H/E* level of 0.067–0.097 and *H/E* level of 0.074–0.107, whereas the DCMS-prepared Zr–Si–N films exhibited a low H/E* level of 0.063–0.087 and *H/E* level of 0.070–0.097 accompanied with lower hardness values of 11.7–23.6 GPa. Musil [39] proposed that hard nanocomposite films with H/E* > 0.1 and *W_e_* ≥ 60% exhibited high toughness. The Zr_60.2_Si_3.8_N_36.0_ films prepared at a bias voltage of −50 V exhibited the highest H/E* level of 0.107 and *W_e_* level of 72% among the surveyed Zr–Si–N films in this study. In the work of Choi et al. [4], the Zr–Si (5.8 atom %)–N films exhibited the maximum hardness, Young’s modulus, and H/E* values of 33 GPa, 265 GPa, and 0.12, respectively, accompanied with the lowest friction coefficient and the best wear resistance. The common characteristics of the films prepared under a substrate bias voltage of −50 V exhibited high H/E*, *H*^3^/E*^2^, and *W_e_* values (Table 2) accompanied by (200) preferred orientation (Figure 5) as is presented in region II of Figure 9b.

**Figure 11:**
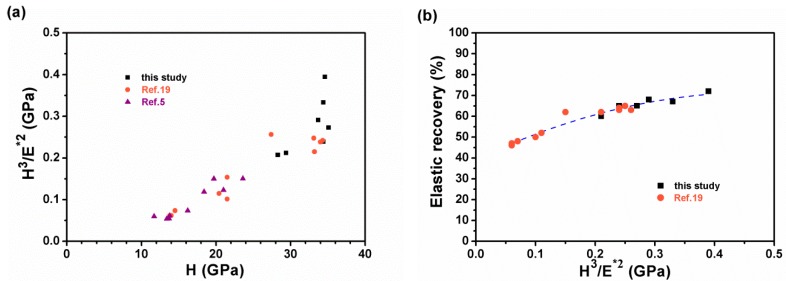
(**a**) *H*^3^/E*^2^ (E*: effective Young’s modulus) as a function of hardness (*H*) and (**b**) elastic recovery (*We*) as a function of *H*^3^/E*^2^ for Zr–Si–N films. (●: reference 19; ▲: reference 5).

The parameters of *H*^3^*/E*^2^ [40,41] and *H*^3^/E*^2^ [28] were widely applied to represent resistance to plastic deformation. Figure 11a presents the relationship between *H*^3^/E*^2^ and *H*, which includes some data from previous studies on Zr–Si–N films [5,19]. Silva Neto et al. [42] reported that the hardness and *H*^3^*/E*^2^ ratio exhibited an increasing trend for the DCMS- and RFMS-cosputtered Zr–Si–N films, and a maximum *H*^3^*/E*^2^ value of 0.40 GPa was accompanied with a hardness of 20.6 GPa. In our previous study [5], DCMS-fabricated Zr–Si–N films prepared without applying a bias voltage exhibited a low *H*^3^/E*^2^ level of <0.15 GPa (*H*^3^*/E*^2^ < 0.18 GPa). By contrast, the HiPIMS-RFMS-cosputtered Zr–Si–N films prepared at a bias voltage of −50, −100, and −150 V exhibited high *H*^3^/E*^2^ levels ranging from 0.21 to 0.39 GPa (*H*^3^*/E*^2^: 0.25–0.48 GPa), whereas the HiPIMS-RFMS-cosputtered Zr–Si–N films prepared at ground state exhibited medium *H*^3^/E*^2^ levels ranging from 0.06 to 0.26 GPa (*H*^3^*/E*^2^: 0.08–0.31 GPa). A high *H*^3^/E*^2^ level of 0.60 GPa was reported for Zr–Si–N films [43]. Figure 11b displays the relationship between *H*^3^/E*^2^ and elastic recovery (*W_e_*). The *W_e_* value increased with an increase in the *H*^3^/E*^2^ value. 

## 4. Conclusions

The effects of ion bombardment during sputtering deposition were enhanced by applying a negative bias voltage, which resulted in an increase in residual stress and a decrease in Si content for Zr–Si–N films relative to that prepared at ground state. Applying a moderate substrate bias voltage makes the Zr–Si–N films a dense structure with high mechanical properties. The hardness exhibited a linear relationship with residual stress ranging from −0.2 to −4.5 GPa and maintained an almost constant level with residual stress ranging from −4.5 to −6.4 GPa, whereas at residual stress above −6.8 GPa, the hardness varied inconsistently. The films prepared under a substrate bias voltage of −50 V exhibited high *H*, H/E*, *H*^3^/E*^2^, and *W_e_* values accompanied with (200) preferred orientation. By contrast, the films prepared at a substrate bias voltage of more than −100 V exhibited an Si-less content, a strong (111) texture, and a high residual stress ranging from –6.8 to –8.8 GPa. Further research should focus on applying the HiPIMS-RFMS cosputtered Zr–Si–N films on the diffusion barrier utility for Cu metallization.

## Figures and Tables

**Table 1:** Chemical composition, surface roughness, and residual stress of the Zr–Si–N films prepared with an average high-power impulse magnetron sputtering (HiPIMS) power of 300 W on Zr target and various RF powers on Si target at various bias voltages.

Sample	Bias	Chemical Composition (at.%)				Time	T ^1^	Ra ^2^	Stress
	(−V)	Zr	Si	N	O	(min)	(nm)	(nm)	(GPa)
Batch A, P_Si_ = 30 W									
Zr_61.6_Si_3.2_N_35.2_	0	61.0 ± 0.4	3.2 ± 0.1	34.9 ± 0.4	0.9 ± 0.2	270	932	1.4 ± 0.1	−5.0 ± 0.4
Zr_62.3_Si_1.4_N_36.3_	50	62.2 ± 0.2	1.4 ± 0.0	36.2 ± 0.4	0.2 ± 0.1	300	1007	1.6 ± 0.0	−6.4 ± 0.6
Zr_62.3_Si_0.4_N_37.3_	100	62.3 ± 2.1	0.4 ± 0.0	37.3 ± 2.1	0.0 ± 0.0	300	966	1.7 ± 0.1	−6.8 ± 0.3
Zr_62.2_Si_0.4_N_37.4_	150	62.2 ± 0.6	0.4 ± 0.0	37.4 ± 0.7	0.0 ± 0.1	300	959	1.2 ± 0.3	−8.8 ± 0.3
Batch B, P_Si_ = 40 W									
Zr_56.2_Si_5.6_N_38.2_	0	55.8 ± 0.2	5.6 ± 0.1	37.9 ± 0.3	0.7 ± 0.2	230	847	0.8 ± 0.1	−4.5 ± 0.6
Zr_60.2_Si_3.8_N_36.0_	50	58.7 ± 0.9	3.7 ± 0.1	35.1 ± 0.6	2.5 ± 0.2	220	747	0.5 ± 0.0	−5.7 ± 0.5
Zr_58.9_Si_0.4_N_40.7_	100	58.0 ± 1.0	0.4 ± 0.1	40.1 ± 1.0	1.5 ± 0.2	300	1080	0.8 ± 0.0	−6.8 ± 0.5
Batch C, P_Si_ = 50 W									
Zr_52.2_Si_7.6_N_40.2_	0	52.0 ± 1.1	7.6 ± 0.2	40.1 ± 1.3	0.3 ± 0.1	210	831	3.5 ± 0.1	−2.8 ± 0.1
Zr_57.6_Si_6.3_N_36.1_	50	56.3 ± 1.9	6.1 ± 0.3	35.3 ± 1.8	2.3 ± 0.5	210	747	0.3 ± 0.1	−4.3 ± 0.3
Zr_54.8_Si_0.3_N_44.9_	100	54.0 ± 2.0	0.3 ± 0.0	44.3 ± 2.1	1.4 ± 0.2	265	981	0.6 ± 0.0	−7.1 ± 0.3

^1^ T: Thickness; ^2^ Ra: Surface roughness.

**Table 2:** Mechanical properties of the HiPIMS-RFMS-cosputtered Zr–Si–N films.

Sample	Bias	Stress	*H* ^1^	E* ^2^	H/E*	*H* ^3^ /E* ^2^	*We* ^3^
	(−V)	(GPa)	(GPa)	(GPa)		(GPa)	(%)
Batch A, P_Si_ = 30 W							
Zr_61.6_Si_3.2_N_35.2_	0	−5.0 ± 0.4	34.3 ± 1.0	408 ± 12	0.084	0.24	64
Zr_62.3_Si_1.4_N_36.3_	50	−6.4 ± 0.6	34.4 ± 1.2	350 ± 14	0.098	0.33	67
Zr_62.3_Si_0.4_N_37.3_	100	−6.8 ± 0.3	29.4 ± 0.6	346 ± 10	0.085	0.21	60
Zr_62.2_Si_0.4_N_37.4_	150	−8.8 ± 0.3	28.3 ± 1.5	331 ± 11	0.086	0.21	60
Batch B, P_Si_ = 40 W							
Zr_56.2_Si_5.6_N_38.2_	0	−4.5 ± 0.6	33.2 ± 1.0	413 ± 13	0.080	0.21	62
Zr_60.2_Si_3.8_N_36.0_	50	−5.7 ± 0.5	34.6 ± 0.5	324 ± 8	0.107	0.39	72
Zr_58.9_Si_0.4_N_40.7_	100	−6.8 ± 0.5	34.4 ± 1.0	413 ± 4	0.083	0.24	65
Batch C, P_Si_ = 50 W							
Zr_52.2_Si_7.6_N_40.2_	0	−2.8 ± 0.1	27.4 ± 1.6	283 ± 8	0.097	0.26	63
Zr_57.6_Si_6.3_N_36.1_	50	−4.3 ± 0.3	33.7 ± 0.9	363 ± 6	0.093	0.29	68
Zr_54.8_Si_0.3_N_44.9_	100	−7.1 ± 0.3	35.1 ± 0.4	398 ± 6	0.088	0.27	65

^1^*H*: Hardness; ^2^E*: Effective Young’s modulus; ^3^*We*: Elastic recovery.
